# binomialRF: interpretable combinatoric efficiency of random forests to identify biomarker interactions

**DOI:** 10.1186/s12859-020-03718-9

**Published:** 2020-08-28

**Authors:** Samir Rachid Zaim, Colleen Kenost, Joanne Berghout, Wesley Chiu, Liam Wilson, Hao Helen Zhang, Yves A. Lussier

**Affiliations:** 1 Center for Biomedical Informatics and Biostatistics, University of Arizona Health Sciences, 1230 N. Cherry Ave, Tucson, AZ 85721 USA; 2 The Graduate Interdisciplinary Program in Statistics, The University of Arizona, 617 N. Santa Rita Ave., Tucson, AZ 85721 USA; 3 College of Medicine, Tucson, 1501 N. Campbell Ave, Tucson, AZ 85721 USA; 4 Department of Mathematics, College of Sciences, The University of Arizona, 617 N. Santa Rita Ave., Tucson, AZ 85721 USA; 5 The Center for Applied Genetic and Genomic Medicine, 1295 N. Martin, Tucson, AZ 85721 USA; 6 The University of Arizona Cancer Center, 3838 N. Campbell Ave, Tucson, AZ 85721 USA; 7 The University of Arizona BIO5 Institute, 1657 E. Helen Street, Tucson, AZ 85721 USA

## Abstract

**Background:**

In this era of data science-driven bioinformatics, machine learning research has focused on feature selection as users want more interpretation and post-hoc analyses for biomarker detection. However, when there are more features (i.e., transcripts) than samples (i.e., mice or human samples) in a study, it poses major statistical challenges in biomarker detection tasks as traditional statistical techniques are underpowered in high dimension. Second and third order interactions of these features pose a substantial combinatoric dimensional challenge. In computational biology, random forest (**RF**) classifiers are widely used due to their flexibility, powerful performance, their ability to rank features, and their robustness to the “P > > *N*” high-dimensional limitation that many matrix regression algorithms face. We propose binomialRF, a feature selection technique in RFs that provides an alternative interpretation for features using a correlated binomial distribution and scales efficiently to analyze multiway interactions.

**Results:**

In both simulations and validation studies using datasets from the TCGA and UCI repositories, binomialRF showed computational gains (up to 5 to 300 times faster) while maintaining competitive variable precision and recall in identifying biomarkers’ main effects and interactions. In two clinical studies, the binomialRF algorithm prioritizes previously-published relevant pathological molecular mechanisms (features) with high classification precision and recall using features alone, as well as with their statistical interactions alone.

**Conclusion:**

binomialRF extends upon previous methods for identifying interpretable features in RFs and brings them together under a correlated binomial distribution to create an efficient hypothesis testing algorithm that identifies biomarkers’ main effects and interactions. Preliminary results in simulations demonstrate computational gains while retaining competitive model selection and classification accuracies. Future work will extend this framework to incorporate ontologies that provide pathway-level feature selection from gene expression input data.

## Background

Recent advances in machine learning and data science tools have led to a revamped effort for improving clinical decision-making anchored in genomic data analysis and biomarker detection. However, despite these novel advances, random forests (**RFs**) [1] remain a widely popular machine learning algorithm choice in genomics given their ability to i) accurately predict phenotypes using genomic data and ii) identify relevant genes and gene products used for predicting the phenotype. Literature over the past 20 years has demonstrated [2–9] their broad success in being able to robustly handle the “*P* > > N” high-dimensional statistical limitation (i.e., when there are more predictors or features “***P***” (i.e., genes) than there are human subjects “*N*”) while maintaining competitive predictive and gene selection abilities. However, the translational utility of random forests has not been fully understood as they are often viewed as “black box” algorithms by physicians and geneticists. Therefore, a substantial effort over the past decade has focused around “feature selection” in random forests (**RF**) [5, 6, 10–14] to better provide explanatory power of these models and to identify important genes and gene products in classification models. Table 1 describes methods of existing feature selection commonly used in random forests as either permutation-type measures of importance, heuristic rankings without formal decision boundaries (i.e., no *p*-values) or a combination of both.

**Table 1:** Random forest feature selection methods and their permutation requirements

Permute	Method	***P***-value	Brief description
No	**binomialRF** [15]	**Yes**	**Optimal splitting features’** ***p*-values obtained via one-sided** ***correlated*** **binomial tests**
	EFS [16]	No	Calculates a global score for each feature using 8 different metrics to measure importance and selects features whose score exceeds the median global score
	AUC-RF [17]	No	Iteratively trains a random forest algorithm and removes predictors in a stepwise fashion to maximize an AUC increase
	RFE, dRFE [18]	No	Iteratively trains a random forest (RF) model and drops uninformative features based on a user-defined criterion
	RF-ACE [19]	No	Creates phony variables called “Artificial Contrasts with Ensembles”, and compares how often these sham variables are used over the real ones
	R2VIM [12]	No	Calculates variable importance (VI) and divides by minimum VI to create relative VI, and choose important features based on a pre-selected cutoff
	VarSelRF, geneSrF [5]	No	Iteratively removes worst .20 (or x-percentage) of all features; retrains RF; selects smallest feature set within one set of best models
Yes	Vita [20]	Yes	P-values are calculated based on empirical null distribution of non-positive importance scores that accelerate null distribution estimates
	Perm [20]	Yes	Permutes outcomes (Y) and determines importance based on which features retained a larger importance in *Y*_*original*_ vs. *Y*_*permuted*_
	PIMP [14]	Yes	Permutes outcome and determines features’ priority based on increases in mutual information or Gini errors. A feature’s *p*-values is produced by an importance measure fitted to a distribution
	VSURF [17]	No	Two-step FS algorithm: 1) uses predictor permutations to identify features robust to noise, and 2) refines model by conducting step-forward inclusion of features until error convergence
	Boruta [13]	No	Creates phony predictors by permuting the values of the shadow vars. Runs RF to identify features’ Z-scores. Eliminates features whose Z-score are less than a threshold. Repeats until convergence

Absence of permutations generally decreases substantially computing time. *P*-values provide explicit ranking of features, which enables objective feature thresholding

While the bioinformatics community have been widely using the above-mentioned approaches to feature selection approaches in multi-analyte biomarker discovery [5], two problems have been hampering their impact in biomedicine. First, random-forests implementations are generally computationally expansive and memory intensive, particularly for identifying molecular interactions. In addition, conventional fully-specified RF classifiers remain opaque to human interpretation, yet there is an increasing consensus among clinicians and machine learning experts that ethical and safe translation of machine learned algorithms for high stake clinical decisions should be interpretable and explainable [21–24].

We hypothesized that a binomial probabilistic framework for feature selection could both improve the computational efficiency of RF classifiers and unveil their otherwise hidden variables for increasing their review and usability by domain experts. We propose the ***binomialRF*** feature selection algorithm, a wrapper feature selection algorithm that identifies significant genes and gene sets in a memory-efficient, scalable fashion, with explicit features for biologists and clinicians. Building upon the “inclusion frequency” [25] feature ranking, binomialRF formalizes this concept into a binomial probabilistic framework to measure feature importance and extends to identify K-way nonlinear interactions among gene sets. The results and evaluation of the simulation, numerical and clinical studies are presented in Section 2. The Discussion and conclusion and presented in Sections 3 and 4, respectively, and the proposed method is formulated in Section 5.

## Results

The simulation and numerical studies used to evaluate the techniques are listed and reviewed in this section. The results and analyses are organized by memory and computational efficiency (Section 2.1), followed by feature selection accuracy and false discovery rates (Section 2.2–2.3) in the simulations and proceeds to detail the numerical studies using the Madelon benchmark (Section 2.4) and the clinical validations from the TCGA repository (Section 2.5) examining breast and kidney cancers.

### Memory efficiency and runtime analysis

To measure memory gains and computational efficiency, two different analyses were conducted in these simulation studies. The first was a theoretical analyses of memory requirements for interaction detection in simulated genomes with 100, 1000, and 10,000 genes. These are clearly smaller than the human genome but serve to illustrate the drastic combinatoric efficiency gained in small dimensional settings. In Table 2, the analyses show the memory efficiency attained by binomialRF to detect 2-way and 3-way interactions. As shown, it can require as much as 170,000 times less memory to calculate 3-way interactions with binomialRF as compared to a classical RF in a moderately large dataset with 1000 variables, potentially impacting memory requirements of grid computers. Note that in linear models, efficient solution paths for $\otimes {X}_{i=1}^K$ only exist for *K* ∈ {1, 2} (LASSO [26] for *K* =1 and RAMP [27] for *K* =2). For *K* > 2, to our knowledge, no algorithm guarantees computational efficiency. In RF-based feature selection techniques, the majority of the techniques requires one to explicitly multiply interactions in order to detect them.

**Table 2:** BinomialRF improves the memory requirements

Features dimension	Interaction order	Memory requirements for interactions		Memory efficiency
		*binomialF*	*Other methods of* Table 1	
10	2	N × 10	N × 55	**~ 5**
	3		N × 175	**~ 17**
100	2	N × 100	N × 5050	**~ 50**
	3		N × 166,750	**~ 1700**
1000	2	N × 1000	N × 500,500	**~ 500**
	3		N × 166,667,500	**~ 170,000**

The improvement is on the orders of magnitude in 2-way and 3-way interactions when compared to other methods of Table 1. One advantage of the binomialRF algorithm is that it can screen for sets of gene interactions in a memory efficient manner by only requiring a constant-sized matrix whereas the current state of the art requires the predictor matrix to increase in size in a combinatoric fashion to screen for interactions. Memory efficiency is defined by $$ \raisebox{1ex}{$\mathrm{Dim}\ \left(\otimes {X}_{i=1}^K\right)$}\!\left/ \!\raisebox{-1ex}{$ Dim(X)$}\right. $$, and interaction memory requirements are defined by the number of columns required to map all k-way interactions

**Fig. 1:**
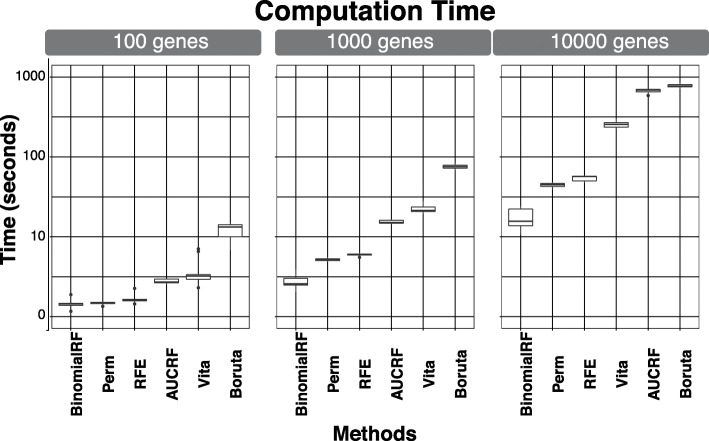
BinomialRF showing substantially improved computational time. The simulation runtimes are measured in seconds and are plotted in powers of ten to show the difference in magnitudes of computation time. The simulation scenarios are detailed in Section 2.1, where the length of the coefficient vector, β varies from 10 to 100 and 1000 features. All simulations were conducted on a 2017 MacBook Pro with 3.1 GHz Intel Core i5 and 16 GB of RAM. All simulations resulted in the binomialRF being the fastest

To compare each algorithm’s runtime, we strictly measure the time for the algorithm to produce its feature ranking and omit other portions using the base *system.time* R function. This runtime is measured in seconds. The boxplot in Fig. 1 displays the range of runtimes (measured in seconds) and graphs them in incremental powers of 10 (i.e., 10^1^, 10^2^, 10^3^, …) to illustrate the difference in magnitudes. As shown in the rightmost panel (10,000 genes) of Fig. 1, the binomialRF algorithm takes, on average, 16.6 s to run, while Boruta averages 779 s, resulting in a 47-fold increase for conducting the same analysis. The techniques omitted from Fig. 1 all resulted in runtimes larger than Boruta (i.e., at least 20X slower than binomialRF), and several of them were unable to process datasets with 10,000 to 20,000 features.

### Feature selection accuracy in simulations

To measure scalability in the predictor space, 500 random forest objects are grown with 500 trees, using simulated genomes sizes 100, 1000, and 10,000 (Fig. 1). Table 3a illustrates and summarizes the results for the main effects analysis across 32 simulation studies including up to 2000 features. Boruta, EFS, VSURF, and binomialRF all attain high precision, while PERM and AUCRF attain the largest recall, and EFS the lowest test error. To mimic a human genome (≈ 20–25,000 genes), a limited simulation scenario generated a synthetic genome with 10,000 genes. However, several techniques other than binomialRF faced rate-limiting computational and memory challenges, preventing us from conducting a full evaluation. Table 3b summarizes the simulation results for *p* = 10,000 where a total of 100 genes were seeded. In this scenario, Boruta and binomialRF again obtained the highest precision values on average, PERM attained the highest recall. However, PERM labeled nearly half the genome as significant, resulting in a precision value near 0. AUCRF and binomialRF produced the most accurate classifiers, though most techniques operated within a similar accuracy range.

**Table 3:** Simulation results of biomarkers

Model	Precision	Recall	Test error	Model size
3A. Results: 100–2000 features				
AUCRF	0.54 (0.25)	0.74 (0.26)	0.27 (0.1)	8.74 (0.13)
binomialRF	**0.91 (0.13)**	0.37 (0.36)	0.33 (0.13)	81.72 (0.08)
Boruta	0.89 (0.15)	0.41 (0.37)	0.32 (0.13)	63.38 (0.1)
EFS	0.83 (0.16)	0.69 (0.27)	**0.25 (0.1)**	8.66 (0.13)
Perm	0.33 (0.33)	**0.82 (0.18)**	0.30 (0.09)	59.42 (0.1)
PIMP^a^	0.18 (0.36)	0.00 (0.01)	0.35 (0.1)	**1.47 (0.11)**
RFE	0.49 (0.35)	0.61 (0.23)	0.3 (0.08)	250.29 (0.09)
VarSelRF	0.67 (0.24)	0.65 (0.29)	0.27 (0.1)	12.31 (0.12)
Vita	0.46 (0.28)	0.66 (0.29)	0.28 (0.1)	35.44 (0.1)
VSURF	0.86 (0.15)	0.44 (0.36)	0.31 (0.12)	40.95 (0.1)
3B. Results: 10,000 features				
AUCRF	0.17 (0.05)	0.33 (0.05)	**0.41 (0.05)**	215.68 (0.01)
binomialRF	0.51 (0.12)	0.14 (0.12)	**0.41 (0.03)**	28.6 (0.03)
Boruta	**0.72 (0.18)**	0.03 (0.18)	0.47 (0.01)	**4.68 (0.02)**
Perm	0.02 (0)	**0.82 (0)**	0.46 (0.03)	4958.26 (0.03)
RFE	0.03 (0)	0.66 (0)	0.44 (0.04)	1950.11 (0.02)
Vita	0.03 (0)	0.52 (0)	0.45 (0.05)	1954.32 (0.02)

The binomialRF and the algorithms in Table 1 were tested across a range of simulation scenarios (Table 6). Mean (standard deviation) results are shown and ranked according to decreasing F1-score. In 3A, the results for all techniques are shown up to 2000 features. In 3B, the results are shown for a limited simulation scenario with 10,000 features and 100 seeded genes. Only a subset of methods are presented in 3B as the remaining were either unable to process 10,000 features (i.e., induced memory errors) or introduced rate-limiting computational challenges (see Fig. 1). Across both tables, Boruta and binomialRF attain the highest precisions, while PERM the highest recall. More studies are required in high dimensional scenarios to better understand each technique’s behavior. Top accuracies are bolded

^a^Across many runs – the PIMP algorithm resulted in no gene predictions, despite running them using their default parameters, resulting in these low precision and recall values. We varied the parameters with no additional success – so we report these results with an asterisk to note they warrant further investigation

### Pure noise selection rate

To complement the variable precision and recall analyses (and thus FDR), and to better understand how often the binomialRF’s detects random noise in the absence of signal, we ran additional simulations in which none of features were informative (i.e., genes seeded ***β*** =0). Therefore, with an outcome fully independent from the predictors, any selection is based on noise, thus measuring the algorithm’s pure noise selection rate. We ran these analyses using 100, 500, 1000, and 2000 features, and the binomialRF produced – on average – a type I error ranging between 0.5–2%. Future simulations will explore artificial datasets with main effects in absence of interactions to quantify these type I errors.

### UCI ML benchmark data repository

The results for the Madelon dataset show the performance attained by all techniques in a benchmark dataset used to evaluate machine learning algorithms. The results in Table 4 indicate that all techniques attain a similar precision and recall, however, with varying model sizes and run times. PIMP, Boruta, and VSURF all result with the smallest models, while PERM results in the largest model. With regards to runtime, similar to the simulations (see Fig. 1), the binomialRF algorithm runs about 4 times as fast the 2nd fastest algorithm, and about 200 times as fast as the slowest.

**Table 4:** UCI ML madelon dataset validation

Model	Model size	Run time	Precision	Recall
VarSelRF	23 (13)	129 (21)	**0.56 (0.01)**	**0.56 (0.02)**
VSURF	3.5 (1.4)	321 (267)	**0.56 (0.02)**	**0.56 (0.03)**
binomialRF	17.1 (3.9)	**5.6 (2.2)**	0.55 (0.02)	0.55 (0.01)
Vita	13 (5.68)	1007 (1220)	0.55 (0.02)	0.55 (0.02)
Boruta	2 (2)	139 (45)	0.54 (0.03)	**0.56 (0.04)**
Perm	240 (13)	269. (329)	0.56 (0.08)	0.54 (0.01)
AUCRF	31 (30)	33 (7.5)	0.55 (0.04)	0.54 (0.02)
RFE	81 (4.2)	20 (1.4)	0.54 (0.06)	0.54 (0.01)
EFS	20 (8.3)	2617 (2126)	0.53 (0.02)	0.54 (0.02)
PIMP	1.7 (1.3)	482 (128)	0.50 (0.04)	0.50 (0.01)

The algorithms in Table 1 were tested and compared using the Madelon benchmark dataset from UCI (described in Methods). Mean (standard deviation) results are shown and ranked according to decreasing harmonic mean of precision and recall of variables. Top accuracies are bolded

### TCGA clinical validations in breast and kidney cancers

**Fig. 2:**
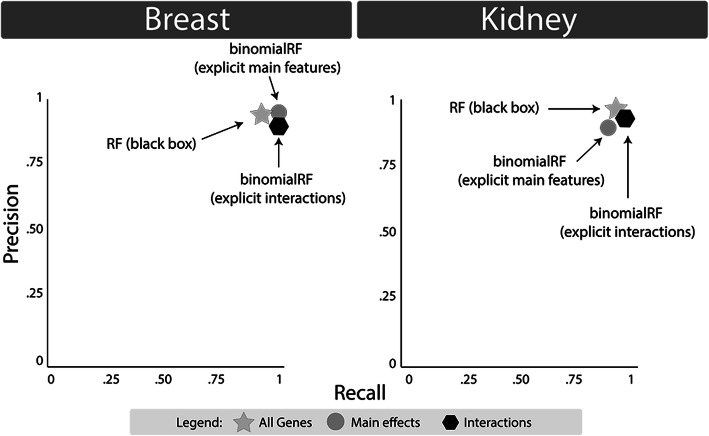
Biomarker accuracies of the TCGA validation study**.** The TCGA validation study was conducted using breast and kidney cancer datasets, accessed via the R package *TCGA2STAT*. The matched-sample datasets were utilized to determine whether binomialRF could produce an accurate classifier via main effects and interactions. Left, the two binomialRF classifiers (51 identified gene main effects; 39 identified gene-gene interactions) and obtained a classifier as accurate as the original black-box RF model with all ~ 20,000 genes. Right, the two binomialRF classifiers (16 identified gene main effects; 11 identified gene-gene interactions) obtained a classifier as accurate as the original black-box RF model with all ~ 20,000 genes

Table 5 shows the results for the breast and kidney cancer TCGA validation studies. The same algorithms from Fig. 1 were included as they were the best suited to analyze high-dimensional datasets. Of note, AUCRF generated memory errors when analyzing the TCGA data and was thus not able to produce results. As demonstrated by prior studies [28], some TCGA datasets are relatively easy classification tasks, as the matched samples are separable, allowing reasonable algorithms to accurately split the samples across the class labels. Therefore, one aspect of value-added in bioinformatics feature selection algorithms is to develop an accurate classifier with a minimal set of genes. In Table 5, Boruta and binomialRF both develop strong classifiers with a small set of genes, however binomialRF provides a more interpretable test statistic, runs about 20X faster, and – as shown in Fig. 2 – extends to detect interactions at no additional cost.

**Table 5:** TCGA dataset validation

Model	Time	Test error	Model size
5A. Breast cancer			
binomialRF	83 (11)	0 (0)	27 (4)
RFE	100 (13)	0 (0)	692 (23)
Perm	112 (16)	0 (0)	1092 (39)
Vita	493 (88)	0 (0)	19,933 (10)
Boruta	1667 (617)	0 (0)	92 (3)
5B. Kidney cancer			
binomialRF	51 (10)	0 (0)	48 (3)
RFE	67 (10)	0 (0)	592 (55)
Perm	73 (12)	0 (0)	867 (55)
Vita	315 (72)	0 (0)	19,760 (41)
Boruta	987 (363)	0 (0)	24 (2)

The algorithms in Table 1 were tested and compared using the TCGA breast cancer and kidney datasets, reporting the mean (and standard deviation in parentheses). Half of the methods were not included as they encountered computation or memory limitations in running the TCGA datasets

Figure 2 illustrates how the binomialRF classifiers, with only 51 genes in breast cancer and 16 in kidney cancer, respectively, obtained comparable performances to that of the highly-accurate black-box classifier with > 19,000 genes results (i.e., precision and recall > 0.98). Furthermore, after identifying key statistical interactions (39 in breast, 11 in kidney), we validated their signal by building a classifier exclusively from them with comparable accuracy.

**Fig. 3:**
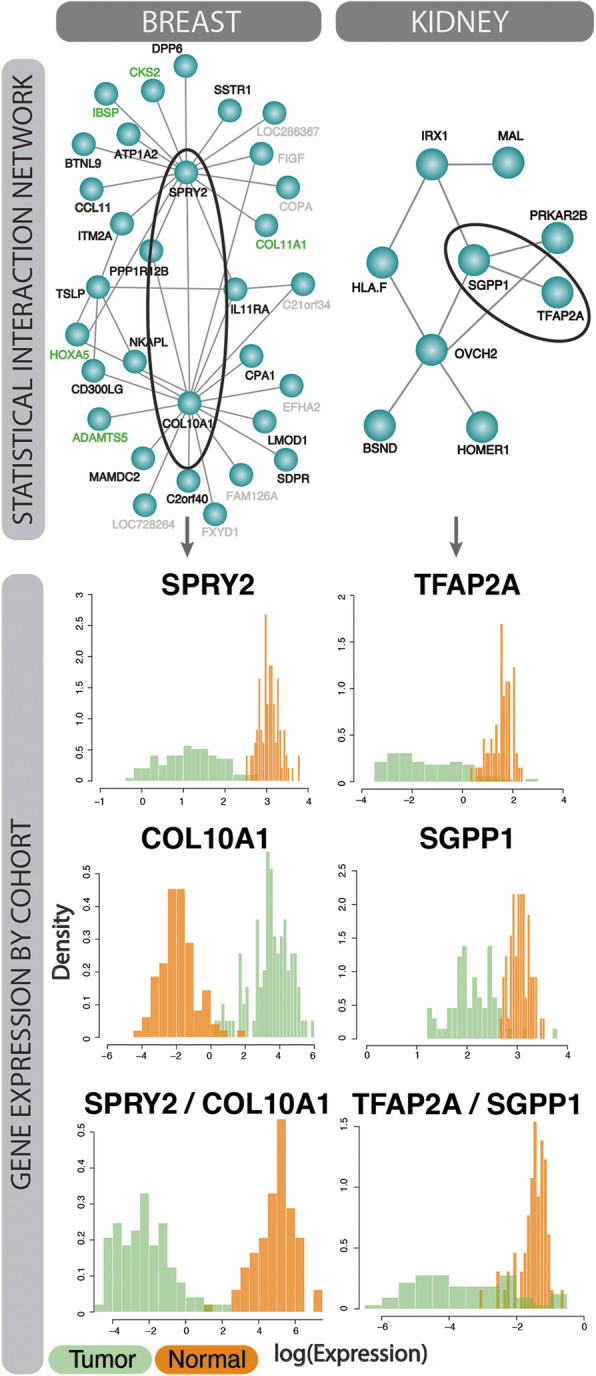
Statistical interactions prioritized by binomialRF in TCGA cancers recapitulate known cancer driver genes. The statistical interaction gene networks (Top) indicate the pairwise biomarker interactions identified by the binomialRF algorithm for the breast (Left) and kidney (Right) cancer datasets. Key features are involved in multiple interactors (super-interactors; e.g., SPRY2; COL10A1). Features names (gene products) found in the literature as associated to cancer pathophysiology are shown in black; those also documented as driving cancer genes in COSMIC are shown in green (Methods); the remainder are grey. Two exemplar statistical interactions (one per dataset) are circled and the log expression of their gene products and of their ratios are shown in the bottom panels. The distribution separation across tumor (green) and normal (orange) cases indicates a potential interaction between these two genes across the cohorts

To validate the identified interactions across both TCGA studies, we constructed networks of their pairwise statistical interactions and assessed whether the log-ratio of the gene expression were distributed differently across tumor and normal samples. Figure 3 provides the statistical interaction networks, as well as exemplar cases of gene-gene interactions in each study. For breast cancer, we present an interaction between SPRY2 and C0L10A1 and for kidney one between TFAP2A and SGPP1. In each study, the two individual genes in isolation are expressed differently across normal-tumor samples indicative of their discrimination power. Further, the log-ratios of both genes show an additional level of statistical signal that is captured from the interaction, suggesting the possibility of biological interaction.

## Discussion

### Numerical studies, RF-based feature selection techniques, efficiency gains, and interactions

The averaged results across all simulation designs are presented in Table 3**,** with the best values of each category bolded, separated into simulations with up to 2000 features (Table 3A) and a set of analyses with 10,000 features (Table 3B) to account for rate-limiting computational and memory challenges introduced by a number of techniques. In low-dimensional numerical studies, techniques such as AUCRF and EFS result in the smallest prediction error, showcasing their strength in the prediction task. The permutation resampling strategy attains the highest recall, which provides users a tool to identify gene products that are potentially relevant for a disease. Boruta, VSURF, and binomialRF algorithms attain the highest precisions (positive predictive value) with reasonable recall. The results in Table 3B illustrate the need to further develop techniques to better operate in high-dimensional scenarios. Attaining a high recall while labeling half the genome as significant is not ideal; on the other hand, attaining a high precision in labeling only a handful of genes might miss some of the biology at play. The techniques in Table 1 do not have a complete grasp of the signal in high-dimensional settings suggesting to a.) continue developing and refining them, and b.) to enrich the analyses at the pathway-level as previous studies have shown that this may facilitate signal detection [29] and introduce a biologically-meaningful dimension-reduction step.

Boruta and binomialRF have very similar performances despite sharing no structural similarities (Boruta builds its selection based on creating phony variables to threshold important ones, while binomialRF models splits via correlated Bernoulli trials). This is likely since both impose a rigid cutoff for selection, resulting in small but highly precise feature sets. However, due to these structural differences, binomialRF runs orders of magnitude faster (see Fig. 1 and Table 4) and can explicitly identify statistical interactions, resulting in computational and statistical advantages**.** The PIMP algorithm with the default parameters resulted in many runs with no feature predictions, demonstrating poor performances. In various additional runs, we modified their function parameters with similar results. binomialRF distinguishes itself with the most optimal memory utilization and runtimes. However, it is worth noting that since the algorithm concentrates its search space in the root of the tree, this strategy of feature selection likely results in attaining higher precision as the algorithm tries to find the features with the largest impact in the decision tree. This trade-off translates to our algorithm missing features with smaller impact that appear further down the tree, resulting in a lower recall, as seen in the simulation studies.

Strobl and Zeileis [30] demonstrate that i) the *Gini importance* (measure of entropy) is biased towards predictors with many categories, and ii) that growing more trees inflates anticonservative power estimates. To address (i), we recommend the user evaluates sets of genes according to their baseline expression levels [31]. For the latter (ii), the binomialRF uses *ntree* parameter (number of trees; Table 6) to calculate a conservative cumulative distribution function (cdf) rather than calculating an anticonservative ***F***_***j***_ (Eq. 1), which mitigates the possibility of overtraining. Our simulations were ran using 500 and 1000 trees with no visible differences across results. We ran five additional simulations (seeding 5/100 genes) using 100, 200, 500, 1000, and 2000 trees to determine the effect of growing more trees. The median results indicate that as the number of trees increases, the metrics tend to converge (data not shown), indicating a stability in the number of trees. For the sampled features parameter, the percentage of features tested in our analyses ranged from 20 to 60%. In addition, for the number of features at each split, we recommend tuning this hyper-parameter via cross-validation. The cross-validated binomialRF function (implemented in our R package) runs a grid-search of equally spaced proportions between 0 and 1 based on the number of folds, and then returns the optimal proportion of features selected for each split.

**Table 6:** Parameters settings for the simulation study

Parameter	Values
Genome size (***P)***	100, 500, 1000, 2000, 10,000
Genes seeded (***β***)	5, 25, 50, 100
Number of trees (***V)***	500, 1000

There are other complementary efforts to improve the efficiency of random forests. Studies [32–35] focus on subspace sampling methods, reducing the search, and ensuring diversity among the features or cases sampled to make the node-splitting process more efficient, rather than biomarker discoveries. Other sets of techniques such as [36] gain efficiency by modifying the learning process. These methods are independent of feature selection and could be combined with any method from Table 1 to further improve RF efficiencies.

binomialRF proposes an automated combinatoric memory reduction in the original predictor matrix (Table 2), while other methods from Table 1 generally require rate-limiting and memory consuming user-defined explicit interactions by multiplying the $\left(\genfrac{}{}{0pt}{}{P}{k}\right)$ interactions. One limitation of assessing memory computation is the inability to conduct a purely theoretical analysis of memory requirements. Further, it is difficult to assess true memory load across different algorithms as some algorithms are serialized while others offer distributed computing across cores. For example, some memory profiling functions in R simply do not function properly in parallel, making such calculations unfeasible. We will continue looking into this in future studies.

Using trees to identify interactions dates back to [37] and partial dependence plots to examine candidate feature interactions. Some algorithms identify sets of conditional or sequential splits, while other strategies (i.e., [37]) measure their effect in prediction error. More recently, works such as [31, 38] look at the frequency of sequence of splits or “decision paths” as a way to determine whether two features interact in the tree-splitting process. For example, iterative random forests (iRF) [38] identify decision paths along random forests and captures their prevalence, therefore benefitting from a combinatoric feature space reduction in the interaction search. Similarly, BART conducts interaction screening by looking at inclusion frequencies of pairs of predictors [31]. Both of these techniques (one in a frequentist and the other in a Bayesian setting) use inclusion frequencies to determine interaction importance and then provide additional tools to provide cutoffs. We extend on these by modeling decision paths (i.e., pairs of splits) as exchangeable but correlated Bernoulli random variables from which we can conduct hypothesis tests. We construct our algorithm on the same principle of using sequence of splits (i.e., decision paths) to identify interactions and extend them by introducing our modeling framework. binomialRF automatically models these sequential split frequencies into a hypothesis testing framework using a generalization of the binomial distribution that adjusts for tree-to-tree data co-dependency. This contribution provides an alternative *p*-value-based strategy to explicitly rank feature interactions in any order with the binomialRF, using a simple modification of a user-determined parameter, *k*. In future studies, we will focus our experiments and numerical analyses to compare techniques that are explicitly designed to identify interactions (i.e., binomialRF and iRF). Future work will also aim to refine and polish interaction detection within the binomialRF framework and extend the preliminary results and techniques.

In future studies, we will extend these analyses beyond random forest classifiers and compare binomialRF against variable selection techniques across other algorithms. For main effects, a future study should consider comparing binomialRF to the L-norm family of penalties in logistic regression (i.e., LASSO and elastic net), as well as importance metrics in tree boosting models and neural networks, and variables selected in SVM algorithms. To assess the efficacy of interactions and biological networks, one possibility is to implement network-based and graph-based family of penalties in logistic regression. These simulation comparisons across other machine and statistical learning algorithms must be carefully designed to not simulate data that would introduce biases nor favor one set of methods over another, which is beyond the scope of the current study. For example, in our simulation studies, the data were generated following a logistic distribution that would biasedly favor a logistic regression over binomialRF. Therefore, a more comprehensive simulation with various generative models is required to adequately compare binomialRF (and tree-based methods) to feature selection in generalized linear models, neural networks, and support vector machines.

Finally, datasets from the UCI and TCGA repositories were used to externally validate the simulations. While the UCI datasets are not novel, they provide reliable benchmarks for the machine learning community to measure against as well as confirmatory power to the results of the simulations. In addition, validations with TCGA labels served as accuracy measurements (Table 5) in a high-dimensional setting (datasets had approximately 20 thousand features). As shown in Table 5, several of the algorithms listed in Table 1 were unable to provide adequate analyses either due to computational or memory limitations, limiting their usability in certain high-dimensional bioinformatics tasks.

### Moving towards interpretable, white-box algorithms

In recent years, there have been substantial efforts to develop more human-interpretable machine learning tools in response to the ethical and safety concerns of using ‘blackbox’ algorithms in medicine [21] or in high stake decisions [22]. A perspective on *Nature Machine Intelligence* [22], the Explainable Machine Learning Challenge in 2018 [39], and other initiatives serve as reminders of the ethical advantages of using interpretable white-box models over blackbox ones. Novel software packages and methods (i.e., [40, 41]) bring elements of ensemble learning and RFs into the linear model space to combine the high accuracy of ensemble learners with interpretability of generalized linear models. Other initiatives such as the *iml* R package [41] provide post-hoc interpretability tools for blackbox algorithms or provide model-agnostic strategies “to *trust* and act on predictions” [42]. These white-box efforts are converging towards producing more explanatory power that improves ethical and safe decision making. Feature selection methods also improve the transparency of machine learning methods. Further, there is a need to develop algorithms that can better illustrate how they identify and rank features. Among feature selection techniques, binomialRF provides more explicit features and their interactions than conventional RF as well as a prioritization statistic. This differs from the majority of other feature selection methods that have been developed for RF, as they do not provide a prioritization among features (Table 1; *p*-value = no). For those that provide *p*-values, they require memory intensive and time-consuming permutation tests.

The feature selection algorithms in Table 1 are designed to take a high-dimensional set of features (i.e., genes in a genome) and recommend or prioritize a small but important subset of them. They do this either via soft or hard decisions (i.e., p-value ranks vs. sets of discovered genes), but do not provide directionality of effect (i.e., harmful v. protective effect), limiting actionability. The binomialRF provides an effect size along with a p-value, providing a small improvement in this direction to make these algorithms more ‘white-box’ and interpretable, but it is still not a fully a white box algorithm. In contrast, novel algorithms, such as TreeExplainer [43], provide great visualization and model-interpretation tools that provide directionality for feature effects by measuring each feature’s contributions to the prediction. However, TreeExplainer differs from the algorithms in Table 1 as it does not provide an automated or decision-boundary-based mechanism to prioritize features. This does not allow for a fair comparison between these methods, resulting in its exclusion from the analysis. Thus, future work should incorporate the interpretive power of new algorithms (such as TreeExplainer) into feature selection, in order to provide a set of prioritized genes as well as the direction of their effect on the outcome.

As recent work by our lab and others have shown, there is a subspace of genomic classifiers and biomarker detection anchored in pathways and ontologies [44–46] that has yielded promising results in biomarker detection using a priori defined gene sets (i.e., GO [47]). Hsueh et al. have explored the subdomain of ontology-anchored gene expression classifiers in random forests [48]. They also discuss alternate statistical techniques available for geneset analyses and paved the way towards RF-based geneset analysis. In future work, we will direct our efforts along this path and extend binomialRF to incorporate gene set-anchored feature selection algorithms that explore pathway interactions.

## Conclusion

We propose a new feature selection method for exploring feature interactions in random forests, binomialRF, which substantially improves the computational and memory usage efficiency of random forest classifier algorithms and explicitly reveals RF Classifier features for human interpretation. The simulation studies and theoretical analyses compared to previous methods have shown that binomialRF attains a substantially improved runtime (between 30 and 300 fold speed reduction) and a combinatoric reduction in memory requirement for interaction detection (a 500-fold and 170,000-fold memory reduction, for 2-way and 3-way interactions in genomes with 1000 genes). Out of the ten techniques, binomialRF is also among the top four most accurate (precision, recall) across large scale simulations and benchmark datasets. In addition, in clinical datasets, the prioritized interaction classifiers attain high performance with less than 1% of the features and produce pathophysiologically relevant features (evaluated via curation and external reference standards). We have released an open source package in R on GitHub and have submitted it to the CRAN (R archive) for consideration.

Machine learning algorithms are increasingly required to explain their predictions and features in human-interpretable form for high stake decision making. Therefore, there is a need for methods that provide explicit white-box-style classifiers with the high accuracy rates otherwise observed in conventional blackbox-style algorithms (e.g., random forests). Among feature selection methods designed for random forests, binomialRF proves to be more efficient and as accurate for exploring high order interactions between biomolecular features as compared to ten published methods. This increased efficiency for exploring complexity may contribute to improving therapeutic decision making, which may address existing machine learning gaps in precision medicine.

## Methods

**Fig. 4:**
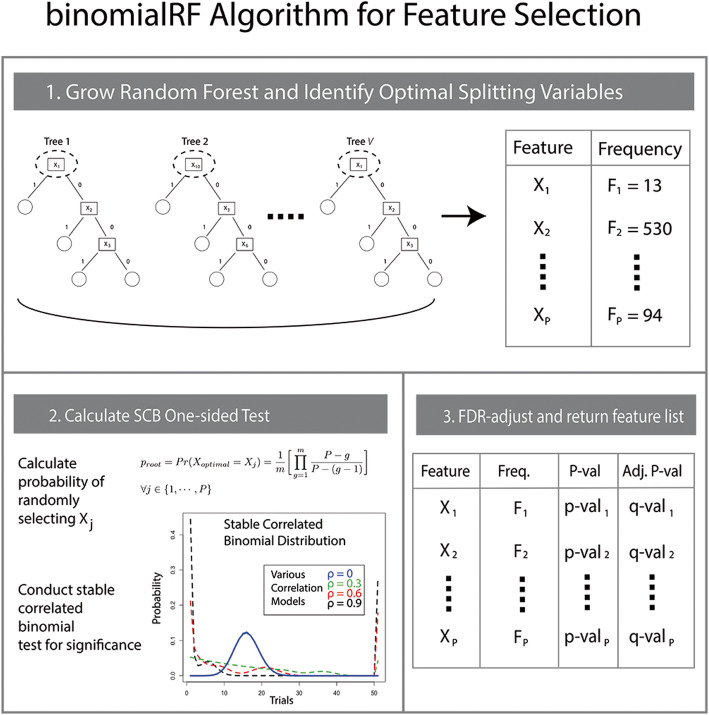
The binomialRF feature selection algorithm. The binomialRF algorithm is a feature selection technique in random forests (**RF**) that treats each tree as a stochastic binomial process and determines whether a feature is selected more often than by random chance as the optimal splitting variable, using a top-bottom sampling without replacement scheme. The main effects algorithm identifies whether the optimal splitting variables at the root of each tree are selected at random or whether certain features are selected with significantly higher frequencies. The interaction-screening extension is detailed in Section 3. *Legend*: *T*_z_ = *z*^*th*^ tree in random forest; *X*_*j*_ = feature j; *F*_*j*_ = the observed frequency of selecting *X*_*j*_; Pr = probability; *P* = number of (#) of features; *V* = # of trees in a RF; m = user parameter to limit *P*; g = index of the product

We propose a new method for feature selection in random forests, binomialRF (Fig. 4), which extends and generalizes the “inclusion frequency” strategy to rank features [25] by modeling variable splits at the root of each tree, ***T***_***z***_***,*** as a random variable in a stochastic binomial process. This is used to develop a hypothesis-based procedure to model and determine significant features. In the literature, there are a number of existing powerful feature selection algorithms in RF algorithms (Table 1). However, this work proposes an alternative feature selection method using a binomial framework and demonstrates its operating characteristics in comparison to existing technology. Table 1 illustrates the advantages of the proposed binomialRF as it is both *p*-value-based and permutation-free, features not identified in our review of literature.

### binomialRF notation and information gain from tree splits

Given a dataset, we denote the input information by, which is comprised of ***N*** subjects (usually < 1000) and ***P*** **features (**genes in the genome; usually *P*≈ 25,000 expressed genes). Genomics data typically represent the “high-dimensional” scenario, where the number of features is much larger than the sample size ***N*** (e.g., “ ***P >  > N*** ”). In the context of binary classification, we denote the outcome variable by ***Y***, which differentiates the case and control groups (i.e., “healthy” vs. “tumor” tissue samples). Random Forests **(RF**) are ensemble learning methods that train a collection of randomized decision trees and construct the decision rule based on combining ***V*** individual trees. We denote a random forest as ***RF =*** {***T***_**1**_, …, ***T***_***V***_}**.** Each individual decision tree, ***T***_***z***_ (z = 1, …, ***V***)*,* is trained by using a random subset of the data and features. This randomization encourages a diverse set of trees and allows each individual tree to make predictions across a variety of features and cases. Each tree only sees ***m < P*** features in the root when it determines the first optimal feature for splitting the data into two subgroups. The parameter, ***m***, is a user-determined input in the random forest algorithm with default values set usually to either $$ \boldsymbol{m}=\sqrt{\boldsymbol{P}} $$ or $$ \boldsymbol{m}=\raisebox{1ex}{$\boldsymbol{P}$}\!\left/ \!\raisebox{-1ex}{$\mathbf{3}\ $}\right. $$. ***F***_***j,z***_ denotes the random variable measuring whether feature *X*_*j*_ is selected as the splitting variable for tree *T*_*z*_ ’s root (Eq. 1):

1 $$ {F}_{j,z}=\left\{\begin{array}{cc}1\kern0.5em ,& if\kern0.5em root\kern0.5em \left({T}_z\right)={X}_j\\ {}0\kern0.5em ,& otherwise\end{array}\right. $$

This results in *F*_*j*, *z*_ following a Bernoulli random variable, *F*_*j*, *z*_ ∼ *Bern*(*p*_*root*_). In **binomialRF**, to test whether the feature *X*_*j*_ is significant in predicting the outcome **Y**, we build a test statistic ${\boldsymbol{F}}_{\boldsymbol{j}}={\sum}_{\boldsymbol{z}=\mathbf{1}}^{\boldsymbol{V}}{\boldsymbol{F}}_{\boldsymbol{j},\boldsymbol{z}}$ to the the null hypothesis of no feature being significant. One would expect that the probability of selecting a feature *X*_*j*_ is equal to that of every other feature *X*_*i*_. Therefore, under the null hypothesis, *p*_*root*_ is constant across all features and trees. Since trees are not independent as they are sampling the same data, *F*_*j*_ follow a ***correlated*** **binomial distribution** that accounts for the tree-to-tree sampling co-dependencies (Fig. 4). The following sections will describe combining the probabilistic framework (2.3), the tree-to-tree sampling co-dependency adjustment (2.4), and the test for significance (2.5).

### Optimal splitting variable and decision trees

**Fig. 5:**
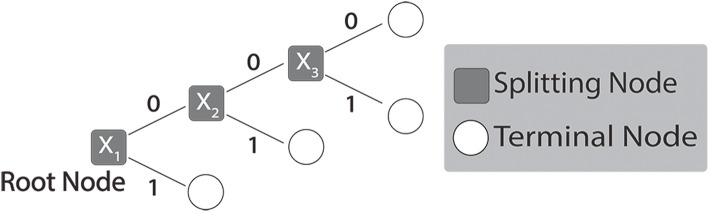
Decision tree and node variables. In the binary split decision tree, *X*_1_ is the optimal splitting feature at the root of the tree, and ${\left\{{\boldsymbol{X}}_{\boldsymbol{j}}\right\}}_{\boldsymbol{j}=\mathbf{1}}^{\mathbf{3}}=\left\{{\boldsymbol{X}}_{\mathbf{1}},{\boldsymbol{X}}_{\mathbf{2}},{\boldsymbol{X}}_{\mathbf{3}}\right\}$ is the optimal splitting sequence that indicates a potential *X*_1_ ⊗ *X*_2_ ⊗ *X*_3_ 3-way interaction, where the symbol “ ⊗ ” denotes interactions

Consider a decision tree, *T*_*z*_, in a random forest (Fig. 5). At the top-most “root” node, *m* features are randomly subsampled from the set of *P* features, and the optimal splitting variable, *X*_*opt*_, is selected as the best feature for separating two classes. Formally, this is stated in Eq. 2.

2 $$ {\mathrm{X}}_{opt}=\mathrm{argma}{\mathrm{x}}_{X_j}\left(\mathrm{Information}\ \mathrm{Gain}\right) $$

Focusing on the root, under a null hypothesis, each feature has the same probability of being selected as the optimal root splitting feature, denoted by *p*_*root*_ = Pr(*X*_*opt*_ = *X*_*j*_) ∀ *j* ∈ {1, …, *P*}. The random variable *F*_*j*, *z*_ (shown in Eq. 1) is an indicator variable that tracks if *X*_*j*_ is selected as the optimal variable for the root at tree *T*_*z*_ . *F*_*j*, *z*_ is a Bernoulli random variable, *F*_*j*, *z*_ ∼ *Bern*(*p*_*root*_). If all trees are independent, summing across trees yields ${F}_j={\sum}_{z=1}^V{F}_{j,z}$ (a binomial random variable). However, trees are not entirely independent since the sampling process creates a co-dependency or correlation across trees.

### Adjusting for tree-to-tree co-dependencies

Each tree in a RF samples *n* ⊂ *N* observations either by subsampling or bootstrapping, which creates a tree-to-tree sampling co-dependency, denoted as ***ρ***. In subsampling, the co-dependency between trees is exactly $$ \rho \le \raisebox{1ex}{$n$}\!\left/ \!\raisebox{-1ex}{$m$}\right. $$, whereas in bootstrapping, the co-dependency is bounded above, i.e., $$ \rho \le \raisebox{1ex}{$n$}\!\left/ \!\raisebox{-1ex}{$m$}\right. $$. Therefore, in all cases, $$ \rho \le \raisebox{1ex}{$n$}\!\left/ \!\raisebox{-1ex}{$m$}\right. $$ provides a conservative upper bound on the co-dependency between trees. This upper bound adjusts for this tree-to-tree sampling co-dependency. Since the number of sampled cases is determined by the user as a RF parameter, the tree-to-tree co-dependency is known and does not require any estimations. Kuk and Witt both developed a generalization of the family of distributions for exchangeable binary data [49, 50] by adding an extra parameter to model for correlation or association between binary trials when the correlation/association parameter is known. We model this co-dependency among trees by introducing either Kuk’s or Witt’s generalized correlation adjustment in the *correlbinom* R package [49], which is incorporated into the binomialRF model.

### Calculating significance of main RF features

At each *T*_*z*_, *m* < *P* features are subsampled resulting in a probability, *p*_*root*_, of *X*_*j*_ being selected by a tree, *T*_*z*_, as shown in Eq. 3:

3 $$ {p}_{root}=1-\left({\prod}_{g=1}^m\frac{P-g}{P-\left(g-1\right)}\left(\frac{1}{m}\right)\right) $$

Using Eq. 3, we can calculate whether *X*_*j*_ provides a statistically significant information gain to discriminate among classes if *F*_*j*_ exceeds the critical value *Q*_*α*, *V*, *p*_, (where *Q*_*α*, *V*, *p*_ is the 1 − *α* ^th^ quantile of a correlated binomial distribution with *V* trials, *p* is the probability of success, and *ρ* correlation). For multiple hypothesis tests, we adjust our procedure for multiplicity using Benjamini- Yekutieli (BY) [51] false discovery rate.

### Calculating significance of RF feature interactions

In classical linear models when detecting 2-way interactions, interactions are included in a multiplicative fashion and treated as separate features with their own linear coefficients. Here, we denote ***X***_***i***_ ***⊗ X***_***j***_ as an interaction between features *X*_*i*_ and *X*_*j*_. One condition imposed in mathematical interaction selection is strong heredity which states that if the interaction *X*_*i*_ ⊗ *X*_*j*_ is included in the model, then their main effects *X*_*i*_ and *X*_*j*_ must be included. Similarly, under weak heredity, at least one of the two main effects must be included in the model if their interaction term is included. In the context of linear models, several existing methods have been proposed to select interactions and studied in terms of their feasibility and utility [52, 53]. Tree-based methods uniquely bypass these conditions as strong heredity hierarchy is automatically induced resulting from the binary split tree’s structure. As Friedman explains, trees naturally identify interactions based on their sequential, conditional splitting process [38]. This “greedy” search strategy reduces the space from all possible, $\left(\genfrac{}{}{0pt}{}{P}{2}\right)$ interactions, to only those selected by trees, greatly reducing computational cost and inefficiencies in identifying interactions. We generalize the binomialRF to model interactions by considering pairs or sets of sequential splits as random variables and modeling them with the appropriate test statistic and hypothesis test.

To modify the binomialRF algorithm to search for 2-way interactions, we add another product term to Eq. 3 denoting the second feature in the interaction set to calculate *p*_2 − *way*_ (Eq. 4).

4 $$ {p}_{2- way}=\frac{1}{2}\left[\ \left(\ 1-\left({\prod}_{g=1}^m\frac{P-g}{P-\left(g-1\right)}\left(\frac{1}{m}\right)\right)\ \right)\left(\ 1-\left({\prod}_{g=1}^m\frac{\left(P-1\right)-g}{\left(P-1\right)-\left(g-1\right)}\left(\frac{1}{m}\right)\right)\ \right)\right] $$

**Fig. 6:**
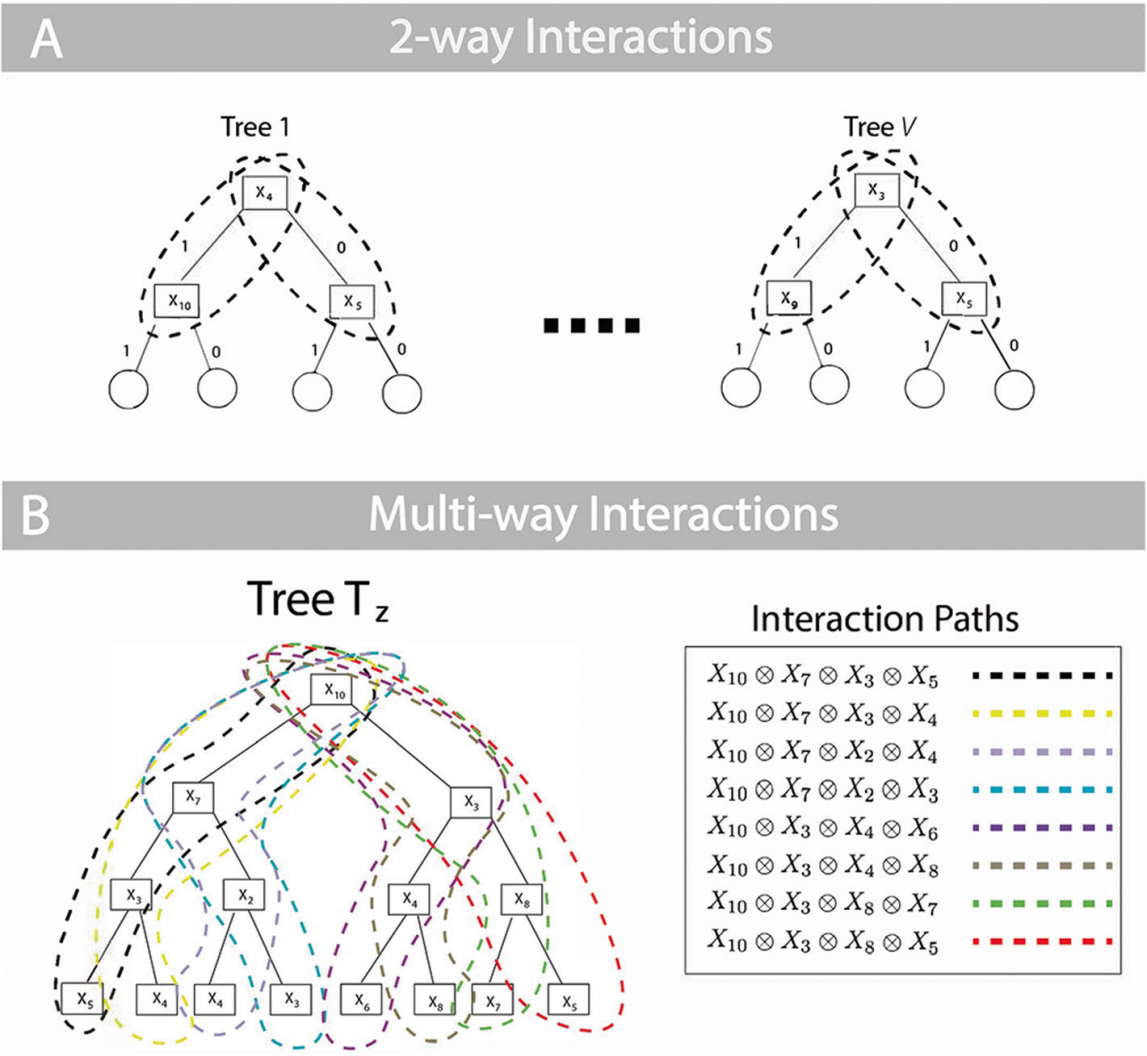
Calculating RF features’ interactions. **a** 2-way Interactions. To extend the binomialRF algorithm for 2-way interaction selection, we define the test statistic which reflects the frequency, *F*_*ij*_ of the pair *X*_*i*_ ⊗ *X*_*j*_ occurring in the random forest. In particular, the probability of an interaction term occurring by random chance is recalculated and normalized by a factor of a half. **b** ***K***-way interactions, ***K*** = 4. Here, we illustrate the tree traversal process to identify all 4-way interactions, $\otimes {\boldsymbol{X}}_{i=1}^4$, with each color denoting a possible interaction path. The legend on the right shows how each interaction path results in a set of 4-way feature interactions. In general, for any user-desired *K*, the k.binomialRF algorithm traverses the tree via dynamic tree programming to identify all possible paths from the *K*-terminal nodes to the root, where ***K***-terminal nodes are all nodes *K*-steps away from the root node

Since we are interested in selecting interactions across variables, if *X*_*j*_ is selected at the root node, then it is no longer available for subsequent selection. Thus, we replace *P* with (*P* − 1). Further, since the interaction can happen two different ways (via the left or right child node), we include a normalizing constant of ½ to account for both ways in which the interaction could occur. Figure 6a illustrates the binomialRF extension to identify 2-way interactions by looking at feature pairs at the root node.

To generalize Eq. 4 into multi-way interactions and calculate *p*_*K* − *way*_, we first note that for any multi-way interaction of size K in a binary split tree results in at most 2^*K* − 1^ terminal nodes. Therefore, there are 2^*K* − 1^ possible ways of obtaining the *K*-way interaction (Fig. 6b). Thus, the normalizing constant in Eq. 4 is replaced with 2^*K* − 1^ in Eq. 5 as a conservative bound on the probability**.** The product of two terms in Eq. 4 is now expanded to the product of *K* terms (each term representing the probability of selecting one individual feature in the interaction set), and (*P* − 1) is replaced with (*P* − *k*) to account for sampling without replacement, which yields Eq. 5.

5 $$ {p}_{K- way}=\frac{1}{2^{K-1}}{\prod}_{k=1}^K\left(\ 1-\left({\prod}_{g=1}^m\frac{\left(P-k\right)-g}{\left(P-k\right)-\left(g-1\right)}\left(\frac{1}{m}\right)\right)\ \right) $$

Next, we update the hypothesis test and modify it to identify 2-way interactions for all possible $\otimes {X}_{i=1}^K$ sets.

### Evaluation via simulations

To understand the strengths and limitations of the binomialRF feature selection algorithm and to compare its performance with state-of-the-art methods, we conduct a variety of simulations and trials against the Madelon benchmark dataset from the University of California – Irvine (UCI), and clinical datasets from The Cancer Genome Atlas (TCGA).

To evaluate each technique’s feature selection accuracy, we measure model size (# of genes discovered), test error, variable precision and recall, and pure noise selection rate. For variable precision and recall, we measure how precise the gene discoveries were and what proportion of the seeded genes in the simulation they captured. Since precision is 1-False Discovery Rate (FDR), variable FDR is implicitly illustrated in Table 3 via the variable precision column, and states how much noise is detected on average relative to the signal detected by the model. The pure noise statistic complements the FDR analysis by analyzing how much pure noise the algorithm detects in absence of a true signal. The five metrics listed above were measured using the equations below:

6-10 $$ {\displaystyle \begin{array}{c}\mathrm{Model}\kern0.5em \mathrm{Size}=\left|\mathrm{Genes}\kern0.5em \mathrm{discovered}\right|,\mathrm{Precision}=\frac{TP}{TP+ FP\hbox{'}}\mathrm{Recall}=\frac{TP}{TP+ FN\hbox{'}}\\ {}\mathrm{Test}\kern0.5em \mathrm{Error}={\sum}_i\left({\overset{\frown }{y}}_i={y}_i\right),\kern0.5em \mathrm{Pure}\kern0.5em \mathrm{Noise}\kern0.5em \mathrm{Selection}\kern0.5em \mathrm{Rate}=\frac{\#\kern0.5em Uninformative\kern0.5em features}{\#\kern0.5em Total\kern0.5em features}\end{array}} $$

These simulation scenarios generate logistically-distributed data to mimic binary classification settings in gene expression data using parameters described in Table 6: genome size = the dimension of the **X** matrix, a coefficient vector ***β*** that denotes the number of genes seeded linked to the outcome, and the number of trees ***V*** grown in the random forest. The parameters used to grow the random forests were V = 500 and 1000 trees, while the number of features selected at each split was set to the default value of 33% (see discussion for additional sensitivity analysis experiments on this parameter). The first two parameters are used to generate the design matrix ***X***_***N × P***_, generate the binary class vector ***Y*** using a logistic regression model.

To determine the performance of binomialRF in detecting important interactions, we conduct a simulation study with 30 total features in which we seeded 4 main effects and all 6 possible pairwise interactions. Since the interactions have to be explicitly multiplied in the design matrix, all techniques except binomialRF had a design matrix with all $30+\left(\genfrac{}{}{0pt}{}{30}{2}\right)$ = 465 features, and the task was to detect all 6 interactions. Since binomialRF can detect interactions from the original design matrix, we used the original matrix with 30 variables first to identify the main effects and then a second time to identify interactions from main effects.

To evaluate computational runtime and efficiency, we measure the theoretical and empirical results of running the feature selection algorithms (Table 1). To measure empirical runtime, 3 simulation studies were run using simulated genomes with 10, 100, and 1000 genes, and we measured their runtime (in seconds) 500 times across each scenario. Figure 1 presents the boxplot of runtimes, measured in seconds and graphed in incremental powers of 10 (i.e., 10^1^, 10^2^, 10^3^, …), to illustrate the difference in magnitudes. To evaluate the theoretical computational efficiency of binomialRF, we compare the theoretical memory requirements of each method described in Table 1 to identify interactions. Since binomialRF can detect interactions using the original design matrix, while other techniques require explicitly mapping the gene-gene interactions, Table 2 compares the memory gain attained across genomes with 10, 100, and 1000 genes when trying to identify 2-way and 3-way interactions.

### Evaluation in UCI benchmark and TCGA clinical sets

To determine the utility of the binomialRF feature selection algorithm in translational bioinformatics, we conduct a validation study using data from the University of California – Irvine machine learning repository (UCI, hereinafter) and from The Cancer Genome Atlas (TCGA; Table 7). The UCI machine learning repository contains over 480 datasets available as benchmarks for machine learning developers to test their algorithms. We present results for all techniques in the Madelon dataset and illustrate their performances using classification accuracy metrics (cases) presented above in Eqs. (6-10). Since true variables are not known in these datasets, variable selection accuracies are not calculated. For the TCGA datasets, we only present results for a subset of the methods that did not encounter memory or computation issues.

**Table 7:** TCGA validation study datasets

Description	Breast cancer	Kidney cancer
**Cohort**	*194 matched tumor-normal samples*	*130 matched tumor-normal samples*
**Outcome prediction**	97 tumor, 97 normal samples	65 tumor, 65 normal samples
**Access**	*TCGASTAT;;getTCGA*	*TCGASTAT;;getTCGA*

We selected the TCGA breast and kidney cancers as two representative datasets with at least 100 matched normal-tumor samples (Table 7). The data were downloaded via the R package *TCGA2STAT* [54], accessed 2020/01, using R.3.5.0. Both RNA sequencing datasets were normalized using RPKM [55] and matched into tumor-normal samples. With many prior studies using the TCGA datasets, our goal was to conduct a binomialRF case study to i) confirm the clinical findings, ii) attain similar prediction performance, and iii) evaluate qualitatively the main effect features and their prioritized interactions. To validate the binomialRF interaction algorithm, we extend the validation of the TCGA datasets *by proposing statistical gene-gene interaction discoveries* and build a classifier from these interactions. We then evaluate their cancer relevance in two ways: (i) a review of literature by trained curators to identify the involvement of these transcripts in cancer pathophysiology, and (ii) a comparison of transcripts with the cancer-driving genes of the COSMIC knowledge-base [56].

### binomialRF implemented as open source package

The binomialRF R package, wrapping around *randomForest* R package [57], is freely available on on CRAN (stable release), with accompanying documentation and help files while experimental updates are released on the Github repository (https://github.com/SamirRachidZaim/binomialRF). The following repository contains all the code and results presented in this manuscript (https://github.com/SamirRachidZaim/binomialRF_simulationStudy).

## Data Availability

The simulated datasets were generated dynamically in the numerical studies and are available in the R scripts in the Github repository, under the code subdirectory which can be accessed via the following link: https://github.com/SamirRachidZaim/binomialRF_simulationStudy/code (see R scripts titled “simulation_XXX.R”). The “Madelon” benchmark dataset was obtained from the UCI Machine Learning repository [https://archive.ics.uci.edu/ml/datasets/Madelon], and the TCGA Breast and Renal Cancer datasets were obtained from the TCGA repository using the *TCGA2STAT* R library. We documented their download and access in our ‘accessTCGA.R’ R script in the Github repository under the TCGA_validation folder (binomialRF_simulationStudy/ code/TCGA_validation). Our open-source *binomialRF* R package is available for installation on CRAN.

## Abbreviations

⊗: Symbol denoting interaction
BY: Benjamini Yekutieli adjustment
cdf: cumulative distribution function
GO: Gene Ontology
RF: Random forest
UCI: University of California – Irvine
TCGA: The Cancer Genome Atlas
iRF: iterative Random Forests
