# Correction to: binomialRF: interpretable combinatoric efficiency of random forests to identify biomarker interactions

**DOI:** 10.1186/s12859-020-03822-w

**Published:** 2020-11-02

**Authors:** Samir Rachid Zaim, Colleen Kenost, Joanne Berghout, Wesley Chiu, Liam Wilson, Hao Helen Zhang, Yves A. Lussier

**Affiliations:** 1 Center for Biomedical Informatics and Biostatistics, University of Arizona Health Sciences, 1230 N. Cherry Ave, Tucson, AZ 85721 USA; 2 The Graduate Interdisciplinary Program in Statistics, The University of Arizona, 617 N. Santa Rita Ave, Tucson, AZ 85721 USA; 3 College of Medicine, Tucson, 1501 N. Campbell Ave, Tucson, AZ 85721 USA; 4 Department of Mathematics, College of Sciences, The University of Arizona, 617 N. Santa Rita Ave, Tucson, AZ 85721 USA; 5 The Center for Applied Genetic and Genomic Medicine, 1295 N. Martin, Tucson, AZ 85721 USA; 6 The University of Arizona Cancer Center, 3838 N. Campbell Ave, Tucson, AZ 85721 USA; 7 The University of Arizona BIO5 Institute, 1657 E. Helen Street, Tucson, AZ 85721 USA

## Correction to: BMC Bioinformatics (2020) 21:374 10.1186/s12859-020-03718-9

Following publication of the original article [1], errors were identified in the References of the Discussion section.

The updated discussion is given below and the changes have been highlighted in **bold typeface**.

## Discussion

Using trees to identify interactions dates back to [37] and partial dependence plots to examine candidate feature interactions. Some algorithms identify sets of conditional or sequential splits, while other strategies (i.e., [37]) measure their effect in prediction error. More recently, works such as [**25**, **58**] look at the frequency of sequence of splits or "decision paths" as a way to determine whether two features interact in the tree-splitting process. For example, iterative random forests (iRF) [**58**] identify decision paths along random forests and captures their prevalence, therefore benefitting from a combinatoric feature space reduction in the interaction search. Similarly, BART conducts interaction screening by looking at inclusion frequencies of pairs of predictors [**25**].

58. Basu, Sumanta, Karl Kumbier, James B. Brown, and Bin Yu. Iterative random forests to discover predictive and stable high-order interactions. Proceedings of the National Academy of Sciences 115, no. 8 (2018): 1943–1948.
